# Survival Probability, Weibull Characteristics, Stress Distribution, and Fractographic Analysis of Polymer-Infiltrated Ceramic Network Restorations Cemented on a Chairside Titanium Base: An In Vitro and In Silico Study

**DOI:** 10.3390/ma13081879

**Published:** 2020-04-16

**Authors:** João P. M. Tribst, Amanda M. O. Dal Piva, Alexandre L. S. Borges, Lilian C. Anami, Cornelis J. Kleverlaan, Marco A. Bottino

**Affiliations:** 1 Department of Dental Materials and Prosthodontics, São Paulo State University (Unesp/SJC), Institute of Science and Technology, São José dos Campos 12245-000, Brazil; amodalpiva@gmail.com (A.M.O.D.P.); alexandre.borges@unesp.br (A.L.S.B.); marco.bottino@unesp.br (M.A.B.); 2 Department of Dentistry, Santo Amaro University (UNISA), São Paulo 04743-030, Brazil; lianami@gmail.com; 3 Department of Dental Materials Science, Academic Centre for Dentistry Amsterdam (ACTA), Universiteit van Amsterdam and Vrije Universiteit, 1081 LA Amsterdam, The Netherlands; c.kleverlaan@acta.nl

**Keywords:** dental implant–abutment design, dental implants, dental materials, finite element analysis, material testing, ceramics

## Abstract

Different techniques are available to manufacture polymer-infiltrated ceramic restorations cemented on a chairside titanium base. To compare the influence of these techniques in the mechanical response, 75 implant-supported crowns were divided in three groups: CME (crown cemented on a mesostructure), a two-piece prosthetic solution consisting of a crown and hybrid abutment; MC (monolithic crown), a one-piece prosthetic solution consisting of a crown; and MP (monolithic crown with perforation), a one-piece prosthetic solution consisting of a crown with a screw access hole. All specimens were stepwise fatigued (50 N in each 20,000 cycles until 1200 N and 350,000 cycles). The failed crowns were inspected under scanning electron microscopy. The finite element method was applied to analyze mechanical behavior under 300 N axial load. Log-Rank (*p* = 0.17) and Wilcoxon (*p* = 0.11) tests revealed similar survival probability at 300 and 900 N. Higher stress concentration was observed in the crowns’ emergence profiles. The MP and CME techniques showed similar survival and can be applied to manufacture an implant-supported crown. In all groups, the stress concentration associated with fractographic analysis suggests that the region of the emergence profile should always be evaluated due to the high prevalence of failures in this area.

## 1. Introduction

Restorations performed using the computer-aided design and manufacturing facility (CAD/CAM) became increasingly popular in dental applications [1]. Due to the wide variety, CAD/CAM materials could be generally divided into two main categories: ceramics and composites [2]. Comparing both categories, indirect composite restorations are more resilient, easier to finish and polish, less abrasive to the antagonist, and allow an easy occlusal adjustment [3]. In contrast, the ceramic restorations present better biocompatibility, superior aesthetics, greater wear resistance and greater color stability [4]. 

Combining the positive properties of CAD/CAM ceramics and composite materials, a hybrid material was developed, which is also known as a polymer-infiltrated ceramic network (PIC) [5]. This material has a relatively low elastic modulus compared to conventional ceramics [6], besides presenting better marginal integrity and machinability [7]. Among the available CAD/CAM blocks from this new class of materials, the Vita Enamic (Vita Zahnfabrick, Bad Säckingen, Germany) stands out for its better long-term color stability [1] and the ability to deform during a load application prior to fracture [8], which ensures proper aesthetics and strength for the rehabilitation. These characteristics are a consequence of the feldspar ceramic involved in a resin matrix [9] based in urethane dimethacrylate (UDMA) and triethylene glycol dimethacrylate (TEGDMA) [5]. The ceramic portion consists of 58–63% of SiO_2_, 20–23% of Al_2_O_3_, 9–11% of Na_2_O, 0.5–2% de B_2_O_3_, and less than 1% of Zr_2_O and CaO [10]. This combination provides adequate wear resistance, flexural strength, and elastic modulus close to dentine tissue [2,11,12]. PICs also have a hardness value between dentin and enamel [11], a maximum fracture load near 2000 N [13], and longitudinal clinical reports with high success rates [14,15]. In observing implant-supported prosthesis, the manufacture of metal–ceramic restorations is defined as the gold standard for prosthetic rehabilitation [16]. However, the monolithic crowns in PIC appear to be a reliable option [16].

Despite the reliability of implant-supported PIC restorations for cemented full-crown design, implant dentistry could present different limitations and aesthetics requirements [17]. Sometimes, the use of a conventional titanium abutment to link the crown and the implant could not be the ideal digital workflow [18]. Since the titanium is a metallic substrate, this abutment can generate a gray zone effect on the peri-implant marginal mucosa [19]. To reduce this effect, the hybrid abutment emerged as an alternative to be used to link the ceramic crown and the implant [20]. The hybrid abutment consists of two parts: a titanium base (Tibase) and ceramic mesostructure. The first one is responsible for keeping the connection between the implant and abutment in metal, and the second one is responsible for improving the peri-implant mucosa aesthetics [20,21,22,23,24,25,26]. For this technique, the crown is indicated to be cemented on the mesostructure (two-piece design).

To manufacture the mesostructure, the ceramic blocks containing a central hole (implant-solution CAD/CAM blocks) are required to allow the connection with the Tibase and the screw access to the implant. The literature reports the possibility of performing mesostructures in zirconia, lithium disilicate [20,25,27] and PIC [22,23,24,28]. The implant solution CAD/CAM blocks also can be machined as a crown without the mesostructure. This approach named one-piece design [29] simplifies the chairside process, requires the use of only one CAD/CAM block, and allows the manufacture of screw-retained crowns [20,28,29]. The comparison of the load-to-fracture of these two designs was performed for zirconia and lithium disilicate restorations [20,26,27]. However, it is well known that dental ceramics fail under fatigue, in consequence of the slow crack growth in stressed areas [30,31]. For this reason, this study investigated the survival probability, Weibull characteristics, and stress distribution of PIC crowns cemented on a chairside titanium base manufactured using different techniques. The null hypothesis was that there would be no difference between the designs for the analyzed parameters.

## 2. Materials and Methods

### 2.1. Specimens Preparations

Seventy-five (75) morse-taper implants (Conexão Sistemas de Prótese, Arujá, SP, Brazil) were installed in polyurethane resin, which is a validated material to simulate the bone tissue in in vitro studies due to its elastic behavior and stiffness [32]. For that, the polyurethane resin was manipulated using an identical volume of base and catalyst homogenized in a rubber bowl. The mixture was poured into (25 × 20 mm) polyvinyl chloride cylindrical support. After the complete resin cure, the polyurethane surface was polished with silicon carbide papers (P600 and P1200) under water cooling in an orbital gridding machine (Buehler, Ecomet 250, Lake Bluff, IL, USA). A sequence of surgical drills was used, according to the manufacturer’s indication (Conexão Sistemas de Prótese, Arujá, SP, Brazil), to make a synthetic surgical alveolus perpendicular to the surface and centered in the polyurethane cylinder. In each cylinder, with the aid of a manual torque wrench, the implants (4.1 × 10 mm) were installed (40 N·cm) keeping 3 mm of the implant not embedded in the resin, following the ISO 14801:2016 for mechanical implant testing. 

All Tibases (Conexão Sistemas de Prótese, Arujá, Brazil) were sandblasted with 50 μm aluminum oxide (Al_2_O_3_) particles at a pressure of 1.5 bar, using an implant analog to assist the laboratorial handling. After, they were cleaned in an ultrasonic bath (5 min with isopropyl alcohol) and received a layer of Alloy Primer (Kuraray Noritake Dental Inc., Okayama, Japan) for 60 s. All titanium bases surfaces were gently blow dried, and the screw access holes were protected with a Teflon tape. A thin layer of titanium dioxide-based powder (Ivoclar Vivadent, Schaan, Liechtenstein) was sprayed onto each of the titanium bases for scanning (inEos Blue, inLab SW4.2, Sirona, Benshein, Germany) and subsequent restorations manufacture. The sets were randomly divided into three experimental groups (*n* = 25), according to the prosthesis manufacturing technique.

#### 2.1.1. Crown Cemented on a Mesostructure (CME) Prosthesis Design: Two-Piece Prosthetic Solution Composed by a Crown Cemented on the Hybrid Abutment

**Figure 1:**
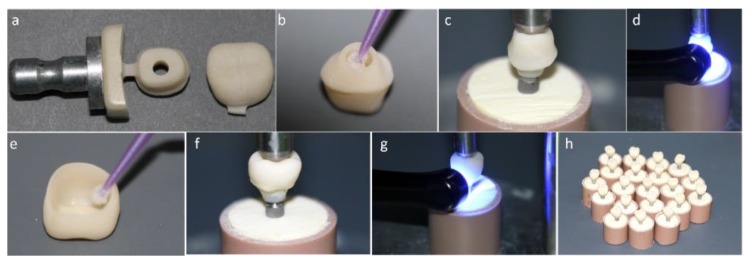
(**a**) Mesostructure machined in implant solution block and monolithic crown machined in conventional block. (**b**) Silanization of the internal surface of the mesostructure. (**c**) Luting of the mesostructure on the titanium base (Tibase) using self-etching resin cement. (**d**) Photoactivation of resin cement. (**e**) Crown silanization. (**f**) Luting of the crown on the mesostructure. (**g**) Light curing. (**h**) Crown cemented on a mesostructure (CME) group crowns completed.

In this technique, the first structure to be manufactured is the mesostructure. For that, the inLab software (Sirona Dental Systems, Bensheim, Alemanha) was used to design the mesostructure and its insertion axis. The data were sent to the equipment Cerec inLab (5884742 D329, Sirona for Dental Systems, Benshelm, Germany), and 25 structures were milled using Vita Enamic IS-14 blocks (Vita Zahnfabrik, Bad Säckingen, Germany) that contain a central access hole. The mesostructures were separated from the remaining blocks with the aid of a diamond blade and fine-grained diamond bur under abundant irrigation. Next, the mesostructures were polished with pink (10,000 rpm) and gray rubbers (8000 rpm) from a Vita Enamic Polishing Set (Vita Zahnfabrick) and cleaned in an ultrasonic bath (5 min in distilled water) to remove debris from the polishing rubbers or surface contaminant that may interfere on the adhesive procedure. To complete the hybrid abutment (mesostructure + Tibase), the mesostructure intaglio surface received a silane agent (Clearfill Ceramic Primer, Kuraray Noritake Dental Inc., Okayama, Japan) for 60 s. The self-etching resin cement (Panavia F 2.0, Kuraray Noritake Dental Inc., Okayama, Japan) was manipulated and applied on the Tibase and the mesostructure, which were held in position with 750 g. The excess cement was removed with a microbrush and light cured (Valo, Ultradent Products, South Jordan, UT, USA) following the manufacturer instructions. The hybrid abutments were installed on the implants with 30 N·cm torque. Next, the mesostructures were scanned for conventional full crown preparations. Then, twenty-five (25) crowns were designed, milled in PIC blocks, polished, and cemented on the hybrid abutment (Figure 1). Both cementation lines were polished with the Vita ENAMIC polishing kit at 5000 rpm.

#### 2.1.2. Monolithic Crown (MC) Prosthesis Design: One-Piece Prosthetic Solution Composed by a Crown Direct Cemented on a Titanium Base

**Figure 2:**
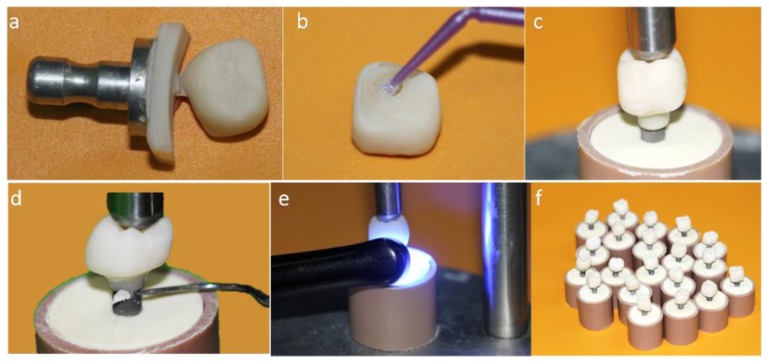
(**a**) Monolithic crown machined in conventional block; (**b**) Silanization of the internal surface of the crown; (**c**) Luting of the crown on the titanium base (Tibase) using self-etching resin cement; (**d**) Removal of excess cement; (**e**) Light curing; (**f**) MC group crowns completed.

In this technique, first, the Tibases were installed on the implants with 30 N·cm torque. Then, the crowns were designed, polished, cleaned, and cemented directly on the Tibases as a conventional abutment. The cementation line was polished with the Vita Enamic polishing kit at 5000 rpm (Figure 2).

#### 2.1.3. MP Prosthesis Design: One-Piece Prosthetic Solution Composed by a Crown Cemented on a Tibase with Screw Access Hole

**Figure 3:**
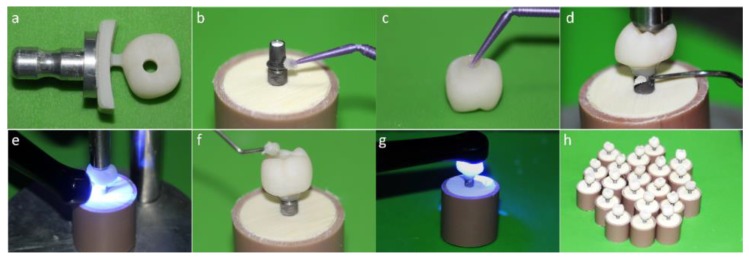
(**a**) Monolithic crown machined in an implant solution block; (**b**) Alloy primer application in the titanium base (Tibase); (**c**) Silanization of the internal surface of the crown; (**d**) Removal of excess cement; (**e**) Light curing; (**f**) Sealing of the screw access hole with composite resin; (**g**) Monolithic crown with perforation; (**h**) (MP) group crowns completed.

For the monolithic crown with perforation (MP) group, the crowns were manufactured in one piece using Vita ENAMIC IS-16 blocks (Vita Zahnfabrik, Bad Säckingen, Germany). The crowns with the occlusal perforation were polished, cleaned, and cemented on the Tibases. Through the screw access hole, the sets (crown and Tibase) were positioned on the implants and with a manual torque wrench fixed with 30 N·cm of torque. For each specimen, the screw access hole was conditioned with 5% hydrofluoric acid for 60 s, washed with a water jet for 20 s, and dried with air jets. Then, the bonding agent was applied, and the access was sealed with composite resin. The cementation line and the composite resin interface were polished with a polishing kit (Vita Zahnfabrik, Bad Säckingen, Germany) at 5000 rpm (Figure 3).

For all groups, the crowns (Vita ENAMIC, Vita Zahnfabrik, Bad Säckingen, Germany) were manufactured with identical anatomy, minimum occlusal thickness at 1 mm, 0.8 mm cervical wall around the base, 0.4 mm cervical terminus, and 80 µm space for the cement layer.

### 2.2. Fatigue Test

**Figure 4:**
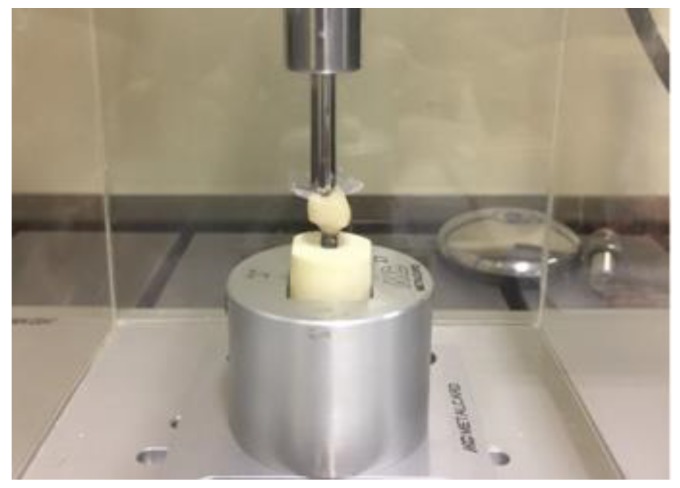
Sample positioned to perform the survival test.

The specimens were stored in distilled water for a period of 24 h prior to the fatigue test. Five samples of each group were submitted to the single load to fracture test (SLF) in an universal testing machine (EMIC DL 1000, EMIC, São José dos Pinhais, PR; 1 mm/min speed, 1000 kgf load cell). From the mean load value (1200 N), the fatigue profile used in the stepwise test was determined. Twenty (20) specimens of each group were tested until failure in an adapted fatigue tester (Fatigue Tester, ACTA, The Netherlands). The fatigue load was delivered (6 mm diameter, stainless steel, water, 25 °C, 1.4 Hz) on the occlusal fossa [13,33]. The test started with 300 N during 5000 cycles (40% of the SLF). After each step of 20,000 cycles, the load was increased [34] in 50 N until the maximum load of 1200 N and 350,000 cycles. The specimens were checked for cracks and/or fractures in each step (Figure 4).

### 2.3. Fractographic Analysis

The failures were classified according to the patterns obtained after the fatigue test [33,34]. To determine the fracture features methodologically, the ceramic fragments were evaluated to identify the direction of crack propagation and location of the origin [33,34,35] with the aid of a stereomicroscope (Stereo Discovery.V20, Carl Zeiss, LLC, USA). For that, the regions of interest were divided into quadrants, and the representative specimens were subjected to photomicrographs of greater magnification in each of these quadrants by scanning electron microscopy (SEM). For this, the specimens were cleaned in an ultrasonic bath with isopropyl alcohol for 10 min, dried, gold sputtered, and analyzed under scanning electron microscopy (SEM; Evo LS15, Oberkochen, Carl Zeiss, Germany) to identify the size and origin of the critical defect. The micrographies were merged to enable the crown fractographic analysis overview. 

### 2.4. Nonlinear Finite Element Analysis 

The three-dimensional (3D) industrial designs of the implant, Tibase, and prosthetic screw were provided by the manufacturer as stereolitographic files (Conexão Sistemas de Prótese, Arujá, Brazil) and imported to the modeling software (Rhinoceros version 5.0 SR8, McNeel North America, Seattle, WA, USA). Then, through automatic reverse engineering, the polygonal models were converted into 3D models formed by NURBS (Non Uniform Rational Basis Spline). In sequence, a cylinder was created corresponding to the in vitro polyurethane cylinder (20 × 25 mm). Then, the implant was centered perpendicularly to the cylinder, containing 3 mm of exposed threads similar to the in vitro test. A Boolean difference was used to ensure the juxtaposition between these structures. The model finished as a volumetric solid containing an implant, Tibase, screw, and fixation cylinder was tripled to obtain three models with identical geometries.

One specimen from each in vitro group was scanned and exported in STL format. The crowns’ 3D models were submitted to the BioCad protocol [36] to perform a volumetric model whose geometry corresponded exactly to the in vitro specimens. Then, this same procedure was repeated to create the mesostructure 3D model. All cement layers were standardized with 80 µm. 

**Figure 5:**
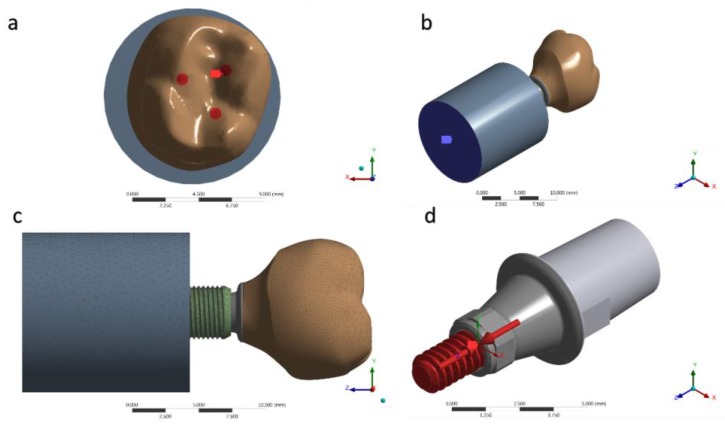
(**a**) Occlusal loading; (**b**) Fixing the system; (**c**) Mesh generated; (**d**) Pre-tension of 30 N·cm.

For a static structural analysis, the models were checked and imported as a STEP file to the analysis software (ANSYS 17.2, ANSYS Inc., Houston, TX, USA). The contacts were considered nonlinear, containing 0.30 μ friction between the structures in titanium [36]. The number of tangent faces between solids was equivalent to assist the analysis convergence. Through an automatic creation, an initial mesh with tetrahedral elements was created; the absence of mesh defined as obsolete by the software was verified prior to final mesh refinement (Figure 5). The 10% convergence test was used to determine the mesh control to ensure the least possible influence on the results of the mathematical calculation [37,38,39]. Each piece of material information was inserted for each solid component in isotropic and homogeneous behavior, requiring the modulus of elasticity and Poisson ratio (Table 1) [5,39,40,41]. During contour definitions, the loading was performed in the occlusal region of the crown. The applied load was 300 N (Figure 5a) on the *Z*-axis [23]. The fixation location was defined under the surface of the polyurethane cylinder, simulating the sample holder in one plane (Figure 5b). A pre-tension was also applied with 30 N simulating the torque (Figure 5d) during the prosthetic screw tightening [42].

The composite resin shrinkage for sealing the screw access hole in the MC group was simulated simultaneously in the analysis using the thermal analogy [43]. The solutions were obtained in total deformation, von-Misses stress, maximum principal stress, and microstrain, for each group. The results were presented on an identical scale of values for visual comparison, as well as the absolute values were plotted on graphs for quantitative analysis of the peaks.

### 2.5. Data Analysis

Survival data were statistically analyzed by Kaplan–Meier and Mantel–Cox tests (Log-Rank and Wilcoxon tests) [44]. Data distribution and reliability analysis were assessed by Weibull analysis associated with two parameters: shape and scale showing the probability distribution of the material to fail in a certain fatigue time using the statistical software (Minitab 16.1.0, State College, PA, USA), with 95% confidence interval. The results obtained in the finite element analysis were exposed and descriptively evaluated through color graphics corresponding to the stress concentration. 

## 3. Results

### 3.1. Fatigue Test

**Figure 6:**
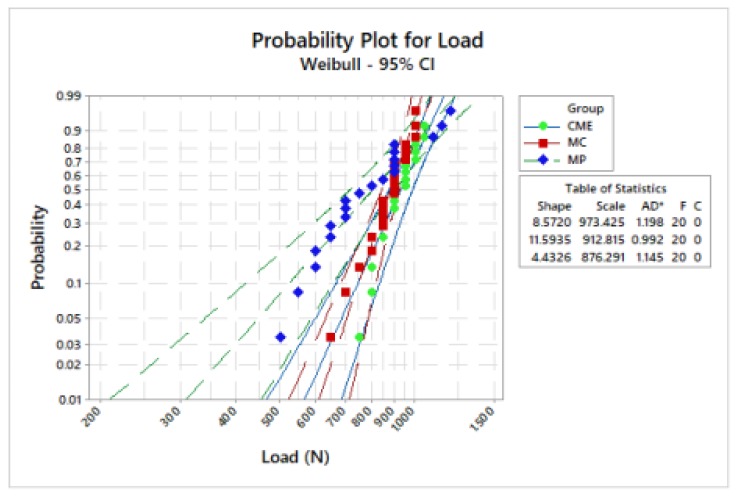
Weibull plot: survival probability versus load (N) reported on sample failure during the fatigue test.

**Figure 7:**
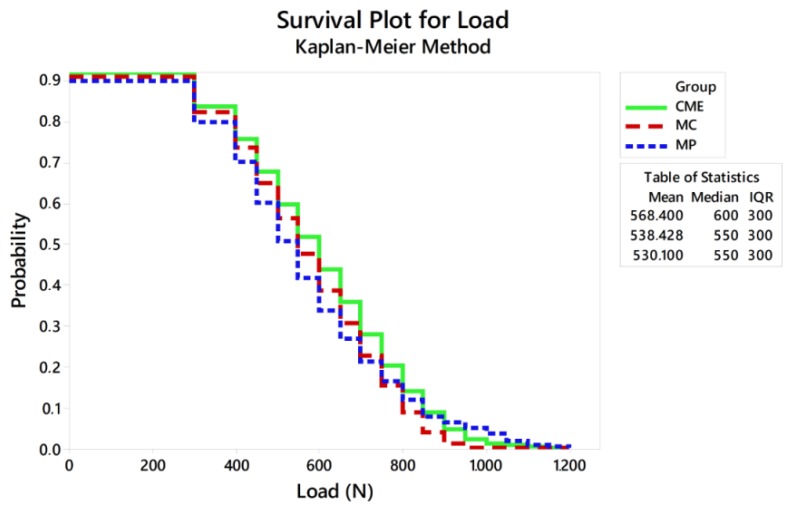
Survival plot using the Kaplan–Meier method.

Weibull analysis showed difference between the mean values of characteristic strength according to the groups (Table 2). Weibull probability plots versus number of cycles reported at the sample failure during the fatigue test are present in Figure 6 and Figure 7. Log-Rank (*p* = 0.17) and Wilcoxon (*p* = 0.11) revealed a similar survival probability between the manufacturing techniques at 300 N and 900 N, according to the confidence interval. However, at 600 N, MP group showed higher survival probability than the MC group, whereas the CME group showed an intermediate behavior (Table 2). 

Regardless of the similar survival and characteristic strength between CME and MP, MP showed lower data variation, being the most reliable technique (Table 3). 

**Figure 8:**
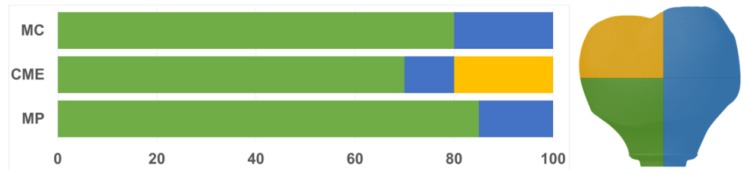
Quantitative analysis of the failures in % regarding the groups and the fracture location. In green, cracks found in the cervical region of the crown. In yellow, cracks found in the occlusal region. In blue, catastrophic failure.

**Figure 9:**
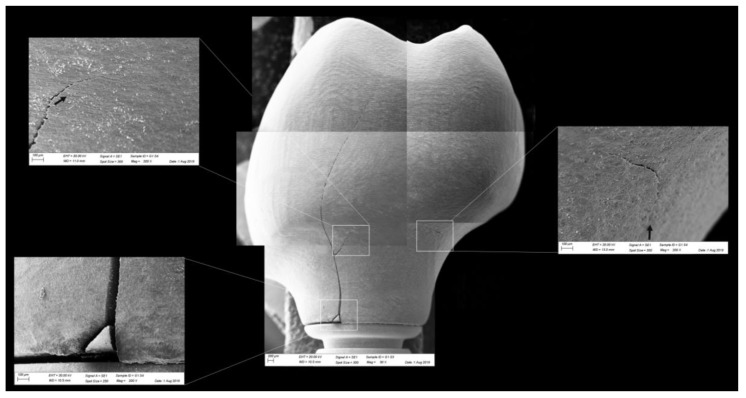
Fractographic analysis of a representative specimen from the MC group. The failure originated in the cervical region and propagated (black arrows) to the top of the restoration without separation of the fractured parts.

**Figure 10:**
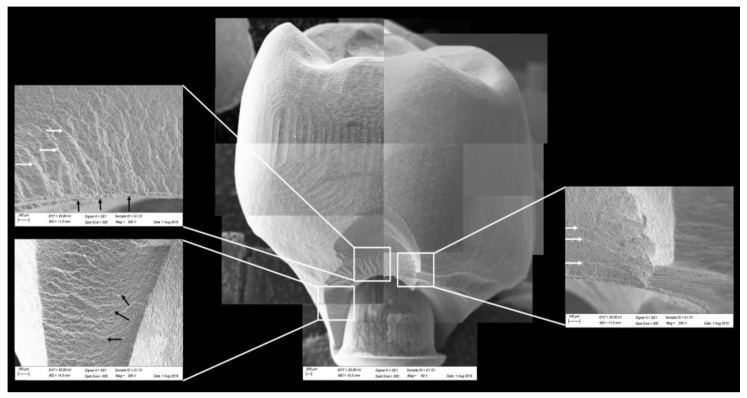
Fractographic analysis of a representative specimen from MP group. The failure originated in the cervical region and propagated to the top of the restoration. The white arrows indicate the hackle lines and the black arrows indicate the twist hackle marks.

**Figure 11:**
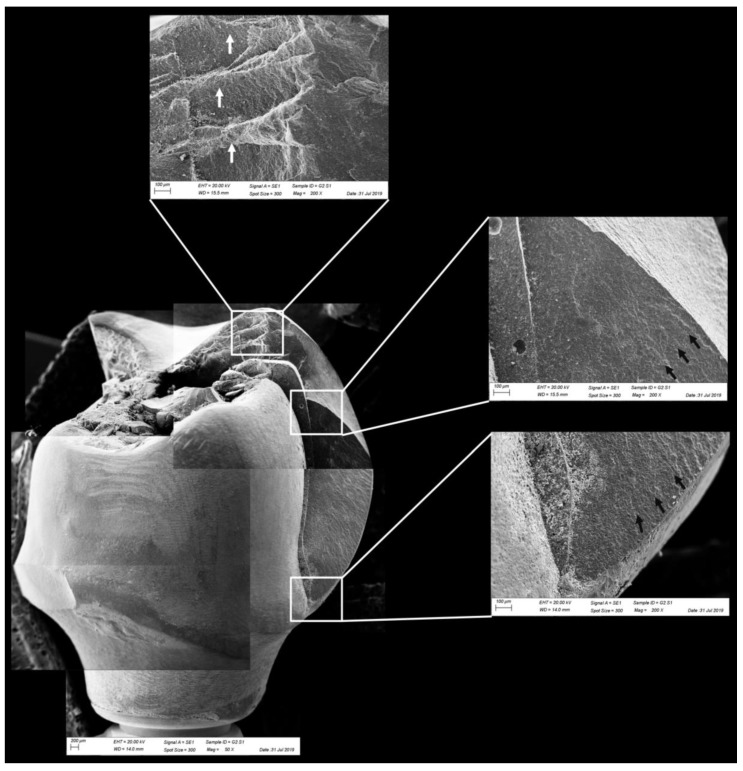
Fractographic analysis of a representative specimen from the CME group. The crown failed without the mesostructure involvement. The white arrows indicate the arrest lines and the black arrows indicate the hackle lines.

**Figure 12:**
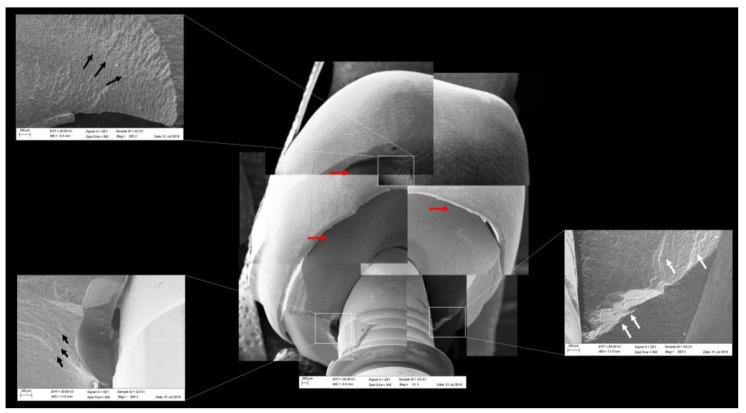
Fractographic analysis of a representative specimen from the CME group. The mesostructure failed without the involvement of the crown. The black arrows indicate the hackle lines, the white arrows indicate the arrest lines, and the red arrows indicate compression curls.

**Figure 13:**
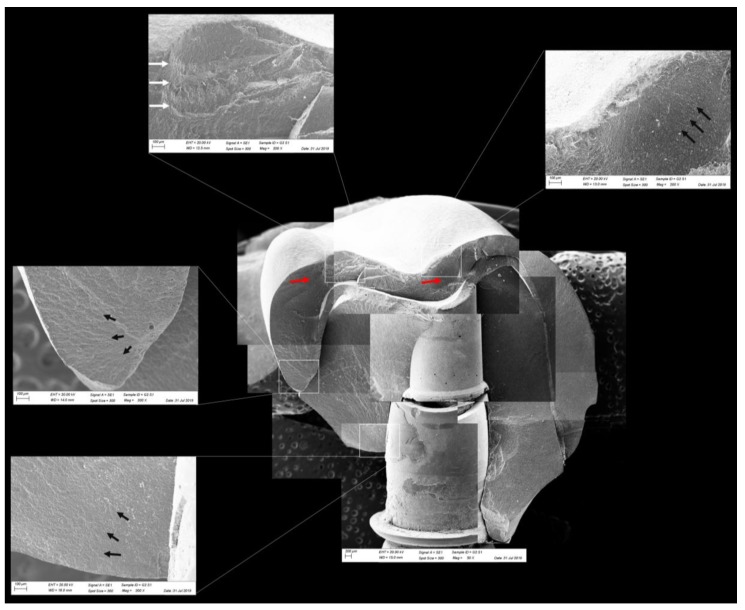
Fractographic analysis of a representative specimen from the CME group. The failure originated in the cervical region and propagated to the top of the restoration. The black arrows indicate the hackle lines, the white arrows indicate the arrest lines, and the red arrows indicate compression curls.

All groups showed cracks, wear facets, and bulk fractures. For the MP group, 15% of the samples presented a bulk fracture in two or more pieces, and 85% of the samples showed chipping failure in the crown emergence profile. For the MC group, 20% of the samples presented a bulk fracture in two or more pieces, and 80% of the samples showed chipping failure in the crown emergence profile. For CME, 20% of the samples showed factures only in the crown exposing the mesostructure, 10% of the samples failed as a bulk fracture with the crack involving the crown and mesostructure, and 70% of the samples failed in the emergence profile without involving the crown (Figure 8). For MC and MP, the fractographic analysis showed that the failure was originated at the cervical area, propagating to the top of the restoration, which was confirmed by the fracture features (Figure 9 and Figure 10). For CME, the specimens in which the fracture was restricted only in the crown, the fractures features suggested the crack propagation direction from the marginal side with several secondary events in the occlusal surface (Figure 11, Figure 12 and Figure 13).

### 3.2. Nonlinear Finite Element Analysis

**Figure 14:**
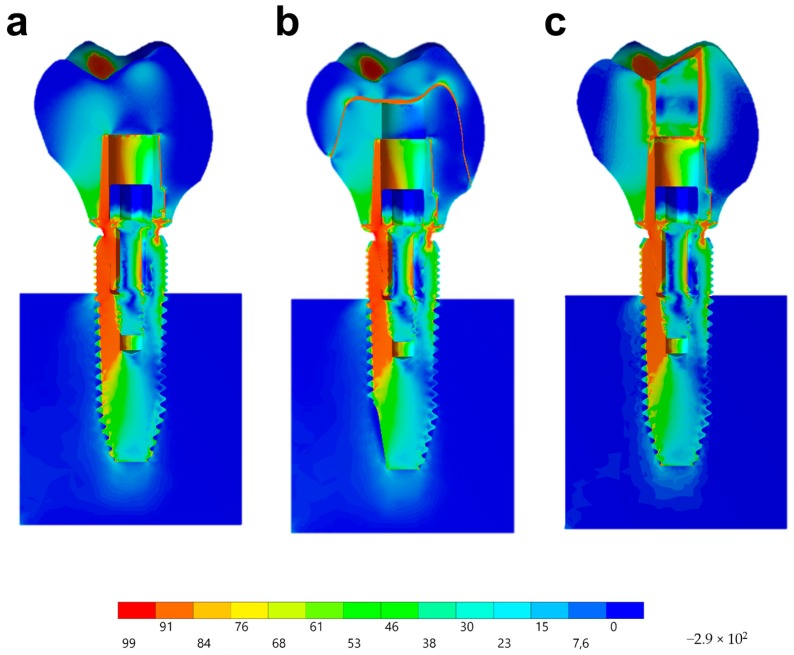
Stress distribution assessed by the von-Mises criterion according to the design of the restoration. (**a**) MC, (**b**) CME, and (**c**) MP.

Observing the von-Mises failure criterion (Figure 14), which demonstrates the total resulting stress in the structures, it was possible to observe that the cervical region was the most involved regardless of the restoration technique, with the composite resin sealing of the group MP presenting a new stress area as well as the cement layer between the crown and mesostructure for the CME group.

**Figure 15:**
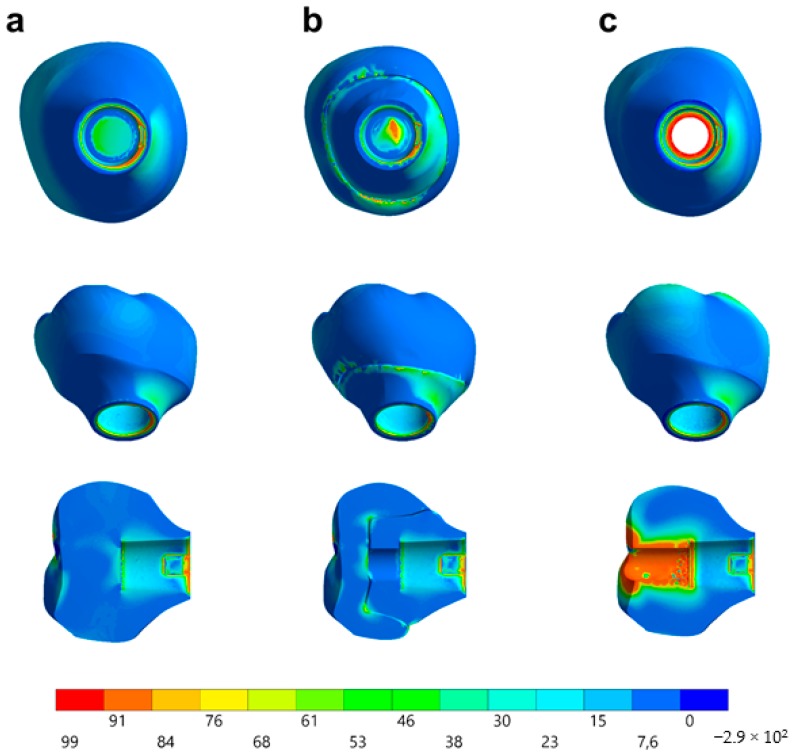
Stress distribution assessed by the maximum principal stress criterion according to the design of the restoration. (**a**) MC, (**b**) CME, and (**c**) MP.

**Figure 16:**
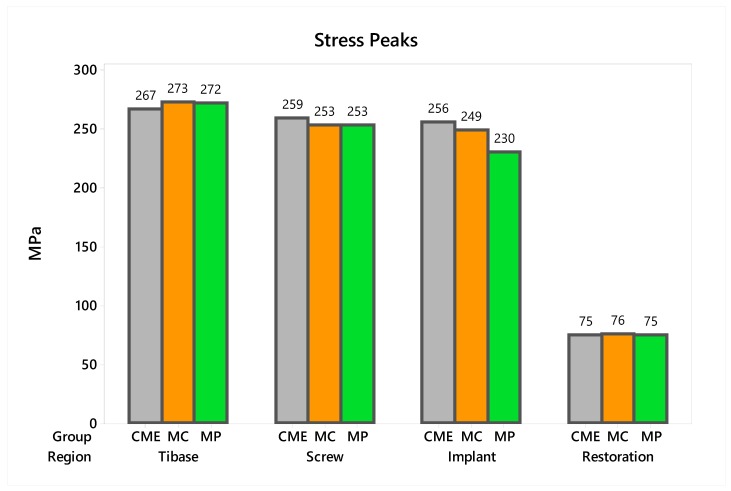
Stress peaks for each structure regarding the different groups. The restoration stress was calculated using the maximum principal stress criterion and the metallic structures using von-Mises criterion.

In observing the tensile stress concentration in the crown, the numerical simulation showed a very similar mechanical behavior between the tested groups (Figure 15), with the highest stress concentration in the cervical region of the crown emergence profile. CME also presented stress concentration on the crown intaglio surface, which is compatible with the failure mode of 20% of the samples during the in vitro test (Figure 8). Furthermore, MP specimens showed high stress concentration in the composite resin used to seal the screw access hole. The stress peaks (Figure 16) corroborate with the colorimetric maps of the results (Figure 14 and Figure 15), not allowing to assume a significant difference between the groups (10%).

## 4. Discussion

This study evaluated the biomechanical behavior and survival probability of implant-supported polymer-infiltrated ceramic (PIC) restorations manufactured using different techniques. The results demonstrated that there is no difference between the groups for the stress distribution and that the reliability was similar between the groups at 300 N and 900 N. Therefore, we partially accept the null hypothesis.

The survival probability of restorations using Tibase as a connection between the implant and the crown is still scarce in the scientific literature [28,45,46]. The indication of this technique depends on the use of CAD/CAM blocks for implant solution that present a connective hole (screw access hole) created by the manufacturer [25,28]. According to the ceramic block size, it is possible to perform a two-piece restoration containing the mesostructure and the crown or a one-piece restoration [15,20,21,26,27,28,46]. These two restorative techniques were simulated in the present study, respectively, for the CME and MP groups.

The use of mesostructure to build the hybrid abutment to replace the conventional titanium abutments has already been reported [21,26,45]. Up to date, in vitro [26,47,48] and in vivo [49] studies and case reports [50,51] reported the use of lithium disilicate or polycrystalline ceramics to manufacture the mesostructure. Meanwhile, one case report [15] and in silico investigations [22,23,24,25] showed the possibility to manufacture the hybrid abutment using PIC material cemented on a Tibase, as performed in this study. Other authors [15] clinically evaluated the CME and MP groups using PIC. The authors suggested that the MP design has a disadvantage in aesthetics compared to the CME due to the presence of the screw access hole filled with composite resin. A literature review [28] suggested the possibility of using PIC to perform a mesostructure; however, the authors affirmed that the lack of longitudinal data does not allow its indication as the material first choice. Based on this, the present study results should assist the clinicians with understanding the biomechanical behavior of this treatment modality.

Comparing the restorative modalities evaluated in the present study, it was possible to observe that there is no difference between CME and MP, which are restorations created using implant solution CAD/CAM blocks and a Tibase. Both designs have already been compared for restorations made with different ceramic material [45]. The authors evaluated 10 anterior lithium disilicate restorations performed for each group and fatigued at 20 Hz until fracture [45], and they found that the MP design was statistically superior. In the present study, 20 restorations were manufactured for each group, the fatigue was adjusted to a maximum of 1.4 Hz, and the survival of MP and CME were not statistically different when PIC is the restorative material. In addition, this study used a posterior total crown with axial loads in order to restrict the failures only in the ceramic material, which was the object of the study.

The literature is not concise regarding the best protocol to use the Tibase. For example, another study [21] compared lithium disilicate anterior crowns using CME and MP techniques. However, the authors only performed the load to fracture using an universal testing machine with *n* = 8 specimens per group. The authors did not find any statistical difference between both designs, corroborating the findings for survival in the present study. Other study evaluated the load to failure of lithium disilicate and zirconia CME versus lithium disilicate MP crowns after aging [27]. Ten specimens per design were submitted to 2000 thermocycles (5–55 °C, water) and mechanical fatigue (150 N, 2 Hz, 100,000 cycles) aging. The authors observed that the lithium disilicate MP presented higher fracture load than a lithium silicate crown cemented on a mesostructure also in lithium silicate or in zirconia. 

Still comparing different designs for using a Tibase, previous research [26] compared CME versus MP made in zirconia and lithium disilicate with *n* = 8/gr. The authors simulated premolars fatigued with 1.2 million thermomechanical cycles associated with 120 N load. After aging, the crowns were tested under an SLF test. Besides the cracks in the ceramic crown, the authors found plastic deformation on the Tibase, screw, and implant. This occurred as a consequence of the static load during compression test, which did not allow the slow crack growth in the ceramic material; this is the mechanism of failure for dental ceramics. For example, if the subject of the study is the ceramic design, it should be ideal that the test could provide the predominance of failures in this region, i.e., it will be possible to understand the restoration weakness and to promote new restorative designs. For this reason, the present study performed the stepwise test to determine at which load step the material has the highest probability of failure.

According to the literature [52], the screw access hole for retrieving ceramic implant-supported screw-retained crowns may decrease their fracture resistance. To prove this statement, the authors tested ceramic crowns in six different groups: monolithic zirconia, veneered zirconia, and lithium disilicate with and without screw access holes. The authors did not use a Tibase; instead, they used a custom abutment and the screw access hole was performed during the crown manufacturing, unlike implant-solution CAD/CAM blocks that already present the perforation. The results of the present study do not agree with these authors [52], because analyzing a ceramic restoration on fatigue, the screw access hole sealed with composite resin does not increase the generated stresses in the cervical region or decrease the restoration characteristic strength. Supporting the results found in the present study, previous authors [20,21,45] reported that groups with screw access holes showed similar load to failure to the groups without access holes. In addition, the authors suggested that perforated crowns may be even more resilient than groups without the access hole. The present study did not find any influence of the access hole on the strength characteristic; however, MP showed higher reliability than MC, corroborating with Roberts and Bailey (2018) [27].

According to a literature review [46] describing the possibilities and limitations of metal-free implant-supported single-tooth restorations, the authors described that one of the main advantages in using a Tibase in a digital workflow is the possibility of performing an adequate emergence profile in the crown. The emergence profile of an implant-supported prosthesis plays an important role in achieving esthetics, which are obtained when the clinician uses a properly concave abutment to mold the soft tissues for an adequate esthetic profile [53]. When improperly designed, the abutment emergence profile will compromise the cervical area blood supply, ultimately resulting in a loss of health and volume of the peri-implant tissues. For that reason, the emergence profile in an implant-supported restoration should be narrow to allow a higher volume of soft tissue [54]. Up to date, there has not been any in vitro study that created a restoration design that presents a concave emergence profile in the cervical area during the hybrid abutment testing [20,21,26,45]. During the present experiment, the 3D designs were created with this concern to simulate the most realistic shape of a restoration surrounded by a well-prepared soft tissue.

Observing the stresses analysis with the finite element method, it is possible to observe that the region of the emergence profile is the area of highest stress concentration in the restoration. This occurred probably due to the increase in the peri-implant soft tissue volume that requires the reduction on the restorative material in that region [15,53,54]. Therefore, the cervical area is the critical area of this restorative modality, since it is located near the fulcrum point of the crown and also because it has the smallest ceramic volume. The finite element method to observe the mechanical behavior of implant-supported single crowns associated with hybrid abutments has previously been reported for studies that compared different combinations of ceramic materials [22,23,24]. Therefore, it has been reported that to reduce the stress in the cervical region of the emergence profile, a flexible material should be used [22]. It is also recommended to use PIC to decrease the stress generated on the crown intaglio surface for CME design [24]. However, the present study demonstrated that MP restorations using only one CAD/CAM block for restoration are very similar to CME groups for the stress concentration in the cervical region. Moreover, the previous studies that simulated this restoration design [22,23,24] were not accompanied by in vitro experiments such as the present study, demonstrating that in fact, the region of stress concentration coincides with the possible failure origin observed in the fractographic analysis. At the occlusal region, the stress difference was caused by the composite polymerization shrinkage between the groups. Despite this, in the in vitro test, the survival was similar between the groups, and the failures were located at the cervical area.

The fractographic analysis followed the recommendations of the ADM (Academy of Dental Materials) guidance [55]. Thus, in a systematic protocol, all fractured restorations were observed under stereomicroscope to identify fracture features that could indicate the crack direction propagation. In this sense, the main failure mode observed was the crack/fracture of the cervical region in the emergence profile, while a smaller percentage of samples failed radially by splitting the crown into two or more pieces. However, for the CME group, different failure modes were also observed due to the presence of a second cement layer (between the mesostructure and the crown). In this group, in addition to failures in the cervical region, some specimens presented only crown fractures without mesostructure involvement. Some specimens presented mesostructure fracture without crown involvement, and two samples allowed the crack to propagate and separate the crown and mesostructure as a monolithic block. These failure modes in CME design have also been observed by previous studies that analyzed these restoration designs with other materials [26,27]. A fractographic analysis performed on failed zirconia mesostructure with Tibase during clinical use showed the same fracture pattern with the direction of crack propagation from the cervical area to occlusal [29], suggesting that the methods of the present study could provide failure modes similar to those found clinically. 

In observing SEM micrographies, it was possible to note that all failures originated from the crown cervical region and spread toward the occlusal region. Secondary effects (damages) in the occlusal third near the compression region were also observed; however, they did not show fracture features that could suggest that the failure could have been originated in this region. It is important that the fractographic analysis is performed dividing each region of interest of the fractured restoration, analyzing each one in higher magnifications. This methodology is widely used to evaluate fractured dental crowns [56], disk-shaped specimens [55], and even for implant-supported crowns with Tibase [29] to assist in understanding how the failure occurred. 

The higher variability of the failure modes observed for CME was reflected in the larger data variability in the Weibull test, increasing the slope of the data distribution line and the Weibull modulus. Therefore, due to the possible different failure modes in this group, it has lower reliability, and therefore its clinical behavior is less predictable. However, its characteristic strength is not inferior to that of the MP and MC groups, which does not contraindicate this design as an option. The Weibull test approach has already been used for studies with dental ceramics [34] and dental implants [16], and it has been used as a statistic method to understand the relationship of data variation and final strength during fatigue [57]. 

Considering the reliability of the restoration itself, the lowest characteristic strength calculated was 789 N, which is about 34% less than the maximum fracture load initially calculated to determine the load variation in the fatigue test. This only reinforces that the slow crack growth during fatigue decreases the ultimate strength of ceramic restorations and should be taken into consideration during the experimental design of the test [16,31]. The Kaplan–Meier curve showed that the survival probability of the restorations decreases as the load increases, being less than 6% at 900 N, regardless of the design. 

For this paper, the MC group simulated the use of a monolithic non-perforated crown as if the Tibase was a conventional abutment for cemented crowns. Although this design survived as well as the CME group and presented an adequate stress concentration, this design does not follow the manufacturer’s recommendation [15]. The Tibase is not indicated as a conventional abutment because of the height of the platform, which cannot allow the complete removal of the resin cement from the cervical area in oral medium [15]. This group was created to elucidate whether the difference between the CME and MP groups would be due to the screw access hole of MP group or, due to the second cement layer on the CME design. No difference was calculated between these groups, suggesting that according to the manufacturer’s recommendation, both techniques can create crowns of equal geometry, and the same clinical indication is correct. It is noteworthy that there was no loss of the composite resin used to seal the screw access of the MP group, which is not uncommon to be observed clinically for metal–ceramic crowns. Further studies evaluating the interface and bond strength between implant solutions CAD/CAM blocks and composite resin should be performed.

## 5. Conclusions

Using a digital workflow, the survival of an implant-supported restoration with PIC does not depend on the technique used to make it. The stress concentrations associated with fractographic analysis suggest that the emergence profile of the restoration should always be evaluated due to the high prevalence of failures in this area.

## Figures and Tables

**Table 1:** Mechanical properties of the materials used in this study.

Material	Elastic Modulus (GPa)	Poisson Ratio	Reference
Titanium	110	0.33	[39]
Polymer infiltrated ceramic	30	0.28	[5]
Polyurethane	3.6	0.3	[40]
Resin cement	18.3	0.3	[41]

**Table 2:** Crowns survival in fatigue at different load missions.

Survival Probability (%)		MP	MC	CME
300 N	Upper bound	88	87	85
	Average	84 ^A^	82 ^A^	80 ^A^
	Lower bound	79	77	74
600 N	Upper bound	50	36	40
	Average	44 ^A^	30 ^B^	33 ^AB^
	Lower bound	37	24	27
900 N	Upper bound	7	6	9
	Average	5 ^A^	1 ^A^	6 ^A^
	Lower bound	2	0.5	3

Similar capital letters correspond to no statistical significance between groups in the same row, according to the confidence interval.

**Table 3:** Weibull modulus (*m*), characteristic strength (σ), and confidence intervals. The statistical differences were determined based on the confidence interval (CI).

Groups	*m* (CI)	σ (CI)
MP	8.5 (6.2–1.6)	973.4 (921.9–1027.7)
CME	4.4 (3.2–6.1)	912.8 (877.2–949.8)
MC	11.6 (8.1–16.4)	876.3 (789.1–973.1)
